# Cancer-associated fibroblasts mediate cancer progression and remodel the tumouroid stroma

**DOI:** 10.1038/s41416-020-0973-9

**Published:** 2020-07-09

**Authors:** Judith Pape, Tarig Magdeldin, Katerina Stamati, Agata Nyga, Marilena Loizidou, Mark Emberton, Umber Cheema

**Affiliations:** 1 Institute of Orthopaedics and Musculoskeletal Sciences, Division of Surgery and Interventional Science, University College London, Stanmore Campus, Brockley Hill, HA7 4LP London, UK; 2 Research Department of Surgical Biotechnology, Division of Surgery and Interventional Sciences, University College London, Royal Free Hospital Campus, Pond Street, NW3 2QG London, UK; 3 Faculty of Medical Sciences, University College London, Bloomsbury Campus Maple House, 149 Tottenham Court Road, W1T 7NF London, UK

**Keywords:** Cancer models, Tumour angiogenesis

## Abstract

**Background:**

Cancer-associated fibroblasts (CAFs) are highly differentiated and heterogeneous cancer-stromal cells that promote tumour growth, angiogenesis and matrix remodelling.

**Methods:**

We utilised an adapted version of a previously developed 3D in vitro model of colorectal cancer, composed of a cancer mass and the surrounding stromal compartment. We compared cancer invasion with an acellular stromal surround, a “healthy” or normal cellular stroma and a cancerous stroma. For the cancerous stroma, we incorporated six patient-derived CAF samples to study their differential effects on cancer growth, vascular network formation and remodelling.

**Results:**

CAFs enhanced the distance and surface area of the invasive cancer mass whilst inhibiting vascular-like network formation. These processes correlated with the upregulation of hepatocyte growth factor (HGF), metallopeptidase inhibitor 1 (TIMP1) and fibulin-5 (FBLN5). Vascular remodelling of previously formed endothelial structures occurred through the disruption of complex networks, and was associated with the upregulation of vascular endothelial growth factor (VEGFA) and downregulation in vascular endothelial cadherin (VE-Cadherin).

**Conclusions:**

These results support, within a biomimetic 3D, in vitro framework, the direct role of CAFs in promoting cancer invasion, and their key function in driving vasculogenesis and angiogenesis.

## Background

### Cancer-associated fibroblasts and tumour growth

The permissive role of the tumour microenvironment in contributing to the process of tumour progression is increasingly recognised.^1^ Within this complex and dynamic stromal response, cancer-associated fibroblasts (CAFs) are of particular interest.^2^ CAFs are highly differentiated and activated fibroblasts that comprise a range of subtypes and phenotypes.^3^ In the healthy colon tissue, resting fibroblasts line the lamina propria adjacent to the epithelium, and pre-cryptal fibroblasts contour the walls of the crypts contributing to tissue integrity.^4^ Some subtypes of CAFs are derived from these local fibroblast populations that appear to reside in the margins of the tumour. Other subtypes may migrate from distant sites, such as the bone marrow (BM), whilst some are speculated to have derived from other cell types that differentiate into CAFs. CAFs are also believed to be (differentiated) cancer cells through the endothelial/epithelial–mesenchymal transition (EMT).^3,4^ Furthermore, mesenchymal stem cells (MSCs) have been thought to be able to differentiate into CAFs and consequently give rise to other stromal cells such as endothelial cells (ECs).^2^ CAFs promote tumour growth^5^ by the overexpression of growth factors, cytokines, chemokines and matrix-remodelling enzymes whilst increasing stiffness of the tumour.^6^ This stiffening in itself can drive tumour growth. Recent work has highlighted the role of stiff tumour tissue on cellular communication network factor 1 (CCN1) regulation in endothelial cells, which enhances melanoma cell–endothelium interaction to promote metastasis through the vasculature.^7^ The reactive stroma is in an inflammatory state and under constant stress, such as oxygen and nutrient deprivation. CAFs induce the tumour macrophage polarisation towards the M2 phenotype, also known as tumour-activated macrophages (TAM),^3^ major orchestrators of cancer-related inflammation.^8^ This process is driven mainly by interleukin-6 (IL-6), which is highly expressed by CAFs.^9^ The key signature of CAFs is the overexpression of alpha smooth muscle actin (αSMA), a contractile stress fibre also expressed by myofibroblasts during wound healing.^10^

A number of “CAF markers” are used to differentiate between normal fibroblasts (NFs) and CAFs. They include fibroblast-specific protein-1 (FSP-1/S100A4), fibroblast-activating protein (FAP), platelet-derived growth factor receptor (PDGFR) and prolyl-4-hydroxylase subunit alpha-1 (P4HA1); however, CAFs are a highly heterogeneous population with various activation states present, which makes them difficult to be chracterised.^3,6^ Initially, CAFs repress tumour growth due to gap junction formation amongst activated fibroblasts, but consequently they pave the way for extracellular matrix (ECM) remodelling and stiffening.^11^ The ECM is remodelled physiologically and chemically during cancer progression due to factors expressed and released by the cancer cells and CAFs. This includes proteases breaking down the ECM through increased covalent cross-linking of collagen fibrils, a process mediated by lysyl oxidase (LOX).^2,12^ This in turn increases interstitial fluid pressure within the tissue, which activates CAFs to upregulate transforming growth factor beta (TGF-β-1)^3^ and matrix metallopeptidases (MMPs), thus promoting and guiding cancer cell tissue invasion.^13^ Stiffness plays a major role in cancer progression, and mechanotransduction of the matrix is required for the generation and maintenance of CAFs.^14,15^ CAFs produce and secrete a number of soluble factors, which stimulate neighbouring stromal cells to secrete further tumour growth-supporting soluble factors.^16^ This cancer-stroma crosstalk recruits immune cells and local vasculature due to CAFs increasingly excreting vascular endothelial growth factor (VEGF).^9^ Overexpression of IL-6 by colorectal cancer (CRC) cells and CAFs drives cytokinetic angiogenesis and further upregulates VEGF secretion through prostaglandin-E2 (PGE-2) mediation.^17^ The recruited vascular networks promote cancer escape from the primary tumour and metastases. Colon CAFs specifically secrete growth factors, like hepatocyte growth factor (HGF), which activates mitogen-activated protein kinase (MAPK) and phosphatidylinositol 3-kinase (PI3K)/AKT pathways responsible for cell survival and invasion of the cancer.^4^

### CAFs in 3D cancer models

The use of CAFs in in vitro 2D and 3D cancer models has been very limited in CRC and not very commonly using patient-derived samples. CAFs cultured in collagen have increased contractility compared with NFs.^12^ Previous approaches have used spheroid formation, basic 2D-invasion assays and microfluidic devices^18^ in order to replicate the tumour stroma. These approaches are limited in their 3D representation of the tumour stroma by lacking vital components, such as vasculature and a clearly defined tumour-stroma margin through the compartmentalisation of cancer mass and stroma. By replicating the tumour-stroma margin, it is possible to study the interplay of different cell populations during cancer progression.

Our approach to engineering a 3D in vitro colorectal cancer model incorporates patient-derived CAFs in the stromal compartment, and allows us to study the patient-specific effect on vasculature formation during cancer growth and progression. This novel approach of modelling cancer–CAF interplay allows us to directly demonstrate the cellular crosstalk between the cancer and stromal cells within a stable and stiff ECM.

We hypothesised that invasion of cancer cells into the stromal compartment is enhanced in the presence of CAFs as compared with normal human dermal fibroblasts (HDFs), our control used for this project. We also studied how the presence of CAFs, and the release of growth factors and cytokines, altered the formation of vascular networks and remodelled pre-existing vascular networks.

## Methods

### CAF isolation and propagation

Primary human colorectal cancer-associated fibroblasts were isolated from tumour tissues acquired from surgeries at the Royal Free Hospital. Patients provided informed consent for tissue donation for research, ethics code: 11/WA/0077. Fresh samples were provided by the pathology team, ensuring that diagnostic margins were not compromised. Tissue was disaggregated using a tumour dissociation kit (Miltenyi Biotec, Bergisch Gladbach, Germany) and grown in fibroblast growth medium 2 (Promocell, Heidelberg, Germany). For the first 72 hours (h), cells were left undisturbed; following that, media changes were done every 48 h in order to isolate the fibroblast cell population. The tissue samples were called T7, T10 and T11 for the first round of successful samples, and T6, T9 and T13 for the second lot of successful samples cultured. Patient-derived CAF samples were then tested for positive vimentin expression and negative CK20 expression, to exclude colorectal epithelial cell contamination, and CD31, to eliminate endothelial cell contamination. Metabolic activity of the first three CAF samples was tested of different cell densities using PrestoBlue^TM^ Cell Viability Reagent (Thermo Fisher Scientific, Loughborough, UK). CAF αSMA and metabolic activity were assessed (supplementary Fig. 1). A range of other general fibroblast and more specific CAF gene markers were also investigated.

### Cell culture

Human colorectal adenocarcinoma cell lines HT29 and HCT116 (both European Collection of Cell Cultures through Sigma-Aldrich, Dorset, UK) were grown in Dulbecco’s Modified Eagle Medium (DMEM) at 1000 mg/L glucose (Sigma-Aldrich, Dorset, UK). Human adult-donor dermal fibroblasts (HDF) (Promocell, Heidelberg, Germany) were grown in 4500 mg/L glucose DMEM (Sigma-Aldrich, Dorset, UK). Human umbilical vein endothelial cells (HUVEC) were grown in Endothelial Cell Growth Medium (both Promocell, Heidelberg, Germany). After isolation, CAF cells were cultured using fibroblast growth medium 2 (Promocell, Heidelberg, Germany). All media were supplemented with 10% foetal calf serum (FCS) (First Link, Birmingham, UK) as well as 100 units/mL penicillin and 100 µg/mL streptomycin (Gibco^TM^ through Thermo Fisher Scientific, Loughborough, UK). All cell types were cultured at 5% carbon dioxide (CO_2_) atmospheric pressure at 37 °C, and routinely passaged in 2D monolayers. HDFs and HUVECs were used at passage ≤5.

### Complex 3D models of cancer (tumouroids)

**Fig. 1:**
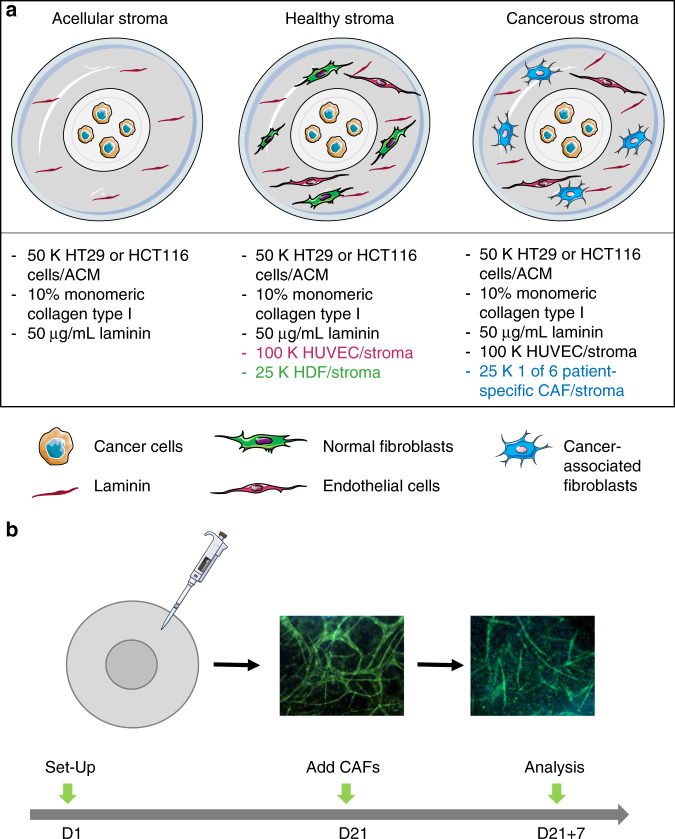
Experimental setups. **a** Birds’-eye view of the three main tumouroid setups used with the respective cellular populations in the ACM and stroma. For all setups, an ACM was nested into a stromal compartment. Both consisted of 10% monomeric collagen type 1 that had undergone plastic compression with the RAFT^TM^ protocol. The stroma was either acellular containing only laminin, healthy, containing HDFs and HUVECs, or cancerous containing one of six patient-specific CAF samples. A schematic was created using Servier Medical Art according to a Creative Commons Attribution 3.0 Unported License guidelines 3.0 (https://creativecommons.org/licenses/by/3.0/). Adjustments and colour changes were made to the original cartoons. (**b**) The “CAF Treatment” set-up. Tumouroids with a normal HDF-containing stroma were left to mature for 21 days to allow endothelial networks to form as previously observed.^19^ After this time, one of three patient-specific CAF samples were applied to the mature tumouroids.

All tumouroids were fabricated using monomeric Type I rat-tail collagen (First Link, Birmingham, UK) and the RAFT^TM^ protocol pages 8–9 (Lonza, Slough, UK) as previously described.^19^ About 10× MEM (Sigma-Aldrich, Dorset, UK) was mixed with collagen and neutralising agent (N.A.) (17% 10 M NaOH (Sigma-Aldrich, Dorset, UK) in 1 M HEPES buffer Gibco^TM^ through Thermo Fisher Scientific, Loughborough, UK, and mixed with cell suspension resulting in 80% collagen, 10% 10× MEM, 6% N.A. and 4% cells. For the artificial cancer masses (ACMs), 5 × 10^4^ cells/ACM of either less-invasive HT29 or highly invasive HCT116 cells were used, and 240 µL of the collagen mix was added to a 96-well plate (Corning^®^ Costar^®^ through Sigma-Aldrich, Dorset, UK). The gel mix was polymerised at 37 °C for 15 minutes (min), followed by plastic compression using the 96-well RAFT^TM^ absorbers (Lonza, Slough, UK). In order to produce “tumouroids”,^20^ the ACMs were nested into a stroma. For the stroma, collagen solution as described above was prepared, and ACMs were directly embedded into a 24-well plate (Corning^®^ Costar^®^ through Sigma-Aldrich, Dorset, UK) containing 1.3 mL of the non-cross-linked collagen mix. Extracellular matrix components were added to this stroma. In this case, mouse laminin^21^ 50 µg/mL (Corning^®^ through Sigma-Aldrich, Dorset, UK) for an acellular stroma additionally to 2.5 × 10^4^ HDFs/CAF samples and 10^5^ HUVECs for a healthy or cancerous stroma, respectively (see Fig. 1a for more detail). The tumouroids were polymerised at 37 °C for 15 min and plastic-compressed using the 24-well RAFT^TM^ absorbers (Lonza, Slough, UK). Tumouroids were cultured for up to 28 days at 5% CO_2_ atmospheric pressure and 37 °C with 50% media changes every 48 h. The media used was a 1:1 mix of the different types used for the cell types within the tumouroids.

### CAF treatment

To study the effect of CAFs on established endothelial networks, CAFs were added to a mature tumouroid containing HDFs and HUVECs in the stroma. A 1.0-mL media suspension containing 2.5 × 10^4^ CAF cells was added to the media mix at day 21 of established tumouroids. CAFs and tumouroids were subsequently left to propagate in co-culture for 7 days with continuing 48 h of 50% media changes. This is additionally demonstrated in Fig. 1b. Investigative measurements were taken at day 21 + 1, day 21 + 3 and day 21 + 7 post CAF addition. *ACTA2* levels were assessed after CAF additiona as an internal control.

### Immunofluorescence

Tumouroids were formalin-fixed using 10% neutrally buffered formalin (Genta Medical, York, UK) for 30 min and then washed and stored in phosphate-buffered saline (PBS) (Gibco^TM^ through Thermo Fisher Scientific, Loughborough, UK). The tumouroids were permeabilised and blocked for 1 h at room temperature using a solution of 0.2% Triton X-100 and 1% bovine serum albumin (BSA) (both Sigma-Aldrich, Dorset, UK) in PBS. Primary antibody incubation was performed overnight at 4 °C followed by three 5-min wash steps with PBS. Secondary antibody incubation was carried out the next day with a 2.5 h incubation at room temperature followed by three 15-min wash steps with PBS. Antibodies were diluted in the same Triton X-100 and BSA solution, and suppliers and source were primary 1:200 anti-CK20 rabbit D9Z1Z (New England Biolabs, Herts, UK), anti-CD31 mouse JC70/A (Abcam, Cambridge, UK), anti-Vimentin mouse V9 (Santa Cruz, Texas, USA) and secondary 1:1000 anti-mouse Alexa Fluor^®^ 488 IgG H&L ab150113 and anti-rabbit DyLight^®^ 594 ab96885 (both Abcam, Cambridge, UK). All tumouroids were counterstained with DAPI, using NucBlue^TM^ (Invitrogen^TM^ through Thermo Fisher, Loughborough, UK).

### Measurement of invasion, endothelial networks and analysis

All tumouroids were imaged using the Zeiss AxioObserver with ApoTome.2 and Zeiss ZEN software (Zeiss, Oberkochen, Germany). In order to measure the invasion from the original ACM into the stromal compartment and the number of endothelial structures, four images were taken at a ×10 magnification evenly spaced out in alignment with a clock face at 12, 3, 6 and 9 o’clock on the same focal plane. This method has previously been described.^19^ The number of endothelial structures was quantified in the same manner with images taken in the same positions but further into the stromal compartment. All samples were assessed for average distance and surface area of invasion and average number of endothelial structures in the stromal compartment. The images obtained were then analysed using the Fiji ImageJ software.^22^

### RNA extraction, cDNa synthesis and real-time PCR

RNA was extracted using the phase separation TRI Reagent^®^ and chloroform method^23^ (both Sigma-Aldrich, Dorset, UK). The total RNA obtained was quantified and assessed for integrity using the NanoDrop^TM^. Transcription into cDNA was conducted using the High-Capacity cDNA Reverse Transcription Kit (Applied Biosystems^TM^ through Fisher Scientific, Loughborough, UK) on the T100^TM^ Thermal Cycler (Bio-Rad, Watford, UK). Primers were designed according to the MIQE with an annealing temperature (Ta) of 60 °C, sequences and efficiencies are listed in Table 1 below and were purchased through Eurofins Genomics (Ebersberg, Germany). Gene target amplification was conducted using iTaq^TM^ Universal SYBR^®^ Green Supermix on the CFX96^TM^ Touch System (both Bio-Rad, Watford, UK) in 10-µL reactions with 20 ng of sample cDNA and primer concentration of 0.2 µM. Relative gene expression was calculated using the ∆Ct and 2^–∆∆Ct^ method^24^ normalising to reference gene *hypoxanthine–guanine phosphoribosyltransferase (HPRT1)* with primers for this gene taken from literature.^25^ Primer design parameters can be found in the Supplementary Section.

**Table 1:** Primer pair sequences, amplicon sizes and efficiencies.

	Primer F′	Primer R′	Amplicon size (bp)	Efficiency (%)
*ACTA2*	*GATAGAACATGGCATCATCAC*	*ACATACATGGCTGGGACATTG*	193	95.1
*S100A4*	*AAAGAGGGTGACAAGTTCAAGC*	*ATGCAGGACAGGAAGACACAG*	182	97.0
*PDGFRA*	*AAGGACTGGGAGGGTGGTCTG*	*TGAAGGTGGAACTGCTGGAACC*	178	98.2
*FAP*	*GTCTTACGCCCTTCAAGAGTTC*	*TCTTGTCCTGAAATCCAGTTTG*	125	98.9
*P4HA1*	*GGGTTGCTGTGGATTACCTG*	*CGAGGCTTGTCCCATTCATC*	178	97.5
*IL-6*	*AGTGAGGAACAAGCCAGAGC*	*GCGCAGAATGACATGAGTTG*	183	96.3
*TIMP1*	*TACTTCCACAGGTCCCACAACC*	*GCATTCCTCACAGCCAACAGTG*	168	93.0
*THBS1*	*GACGGGTTTCATTAGAGTGGTGATG*	*GTATTTCAGGTCAGAGAAGAACAC*	146	97.6
*HIF-1a*	*CCAGCAGACTCAAATACAAGAACC*	*TGTGGGTAGGAGATGGAGATGC*	134	104.0
*MACC1*^19^	*TACGACTCACAAAGCAACAAATGG*	*AAATCATAGGCAGGTTTCCACATC*	100	97.0
*HGF*	*AGCATCATCGAGGGAAGGTGAC*	*CCACGACCAGGAACAATGACAC*	195	109.1
*FBLN5*	*CAAGCCACGACCCGCTACC*	*GCTGCCTCTGAAGTTGATGACAG*	198	104.2
*CDH5*	*CCGTGGTCATCTCAGACAATGG*	*ATCCTCGCAGAAGGTGAACTCG*	107	92.0
*VEGFA*^19^	*GCCTTGCCTTGCTGCTCTAC*	*GAAGATGTCCACCAGGGTCTCG*	155	110.6
*ANG*	*CCTGACCTCACCCTGCAAAGAC*	*GCAAGTGGTGACCTGGAAAGAAG*	142	106.3
*ANPT2*	ACAGGAGGCTGGTGGTTTGATG	GGTTGTGGCCTTGAGCGAATAG	141	110.5
*CTNNB1*	GGGTCCTCTGTGAACTTGCTC	CCGTTTCTTGTAATCTTGTGGCTTG	173	100.6
*PECAM1*	AGAGCCAACCACGCCTCCAG	CACTCCGATGATAACCACTGC	105	108.0
*CXCL8*	TCTTGGCAGCCTTCCTGATTTC	ATTTCTGTGTTGGCGCAGTGTG	172	109.4
*EGFR*^19^	GGCCGACAGCTATGAGATGGAG	AGATCGCCACTGATGGAGGTG	171	97.8
*PLAU*	CGCCACACACTGCTTCATTG	TTTCCACCTCAAACTTCATCTCC	110	108.1
*PTGS2*	CCTCCCACAGTCAAAGATACTCAGG	ACATCATCAGACCAGGCACCAG	115	108.2
*HPSE*^19^	TAAGACCTTTGGGACCTCATGG	CAGATGCAAGCAGCAACTTTGG	193	103.3
*MMP2*	CAGGAGGAGAAGGCTGTGTTC	TAAAGGCGGCATCCACTCG	136	99.4
*MMP7*^19^	ATGAACGCTGGACGGATGGTAG	GGGATCTCCATTTCCATAGGTTGG	140	95.5
*MMP8*	AAGCACACCCAAACCCTGTGAC	TCGACTCTTTGTAGCTGAGGATGC	124	98.4
*MMP19*	GGGATGAGGAAGAAGAAGAGACAG	AAGGGCAGACACTCGGAACAAG	200	99.2
*THBS1*	GACGGGTTTCATTAGAGTGGTGATG	CGTATTTCAGGTCAGAGAAGAACAC	146	97.6
*NES*	CCTTGCCTGCTACCCTTGAGAC	CTGTTTCCTCCCACCCTGTGTC	194	99.9

### ELISA

Media aliquots from cultured tumouroids were taken at every 48 h of media change, kept at −80 °C and analysed for vascular endothelia cadherin (VE-Cadherin) active protein expression using the R&D Systems (Abingdon, UK) Human VE-Cadherin Quantikine ELISA Kit according to the manufacturer’s instructions. The results were read on the Tecan Microplate Reader (Männedorf, Switzerland).

### Protein extraction and western blotting

CAF cell monolayers were lysed for protein with RIPA buffer containing protease inhibitor cocktail at 1:100 dilution (both Sigma-Aldrich, Dorset, UK). Protein content was established using the Pierce^TM^ BCA Protein Assay Kit (Thermo Fisher Scientific, Loughborough, UK). Working solutions were made up to 0.5 µg/µL with RIPA and 2×Concentrate Laemmli Sample Buffer (Sigma-Aldrich, Dorset, UK). In total, 10 µg of protein was loaded onto 10% Mini-PROTEAN^®^ TGX^TM^ Precast 10-well protein gels and run at 200 volts (V) for 45 min using the Mini-PROTEAN Tetra Cell and PowerPac^TM^ 300 in tris-glycine SDS running buffer (all Bio-Rad, Watford, UK). Protein ladder SeeBlue^TM^ Plus 2 pre-stained protein standard (Invitrogen^TM^ through Thermo Fisher Scientific, Loughborough, UK) was used. Dry transfer was conducted using Trans-Blot^®^ Mini Nitrocellulose Transfer Packs and the Trans-Blot^®^ Turbo^TM^ Transfer System (Bio-Rad, Watford, UK). Membranes were blocked for 1 h with 5% milk (Sigma-Aldrich, Dorset, UK) (in tris-buffered saline and 1% Tween 20 (TBST), both Bio-Rad, Watford, UK), incubated with 1° antibodies for α-SMA 1A4 and loading control β-tubulin N-20 in 5% milk overnight at 4 °C at dilutions 1:1000 and 1:200, respectively, followed by five quick and three 5- min washes with TBST. About 2° antibodies IgG-HRP anti-goat sc-2953 and anti-mouse sc-2314 at 1:1000 dilutions were incubated for 1 h in 3% milk (all antibodies through Santa Cruz Biotechnology, Dallas, USA), followed by three 15-min washes and developed using Pierce^TM^ ECL Western Blotting Substrate (Thermo Fisher Scientific, Loughborough, UK). Blots were imaged using the ChemiDoc^TM^ XRS imaging system and Image Lab^TM^ software (Bio-Rad, Watford, UK). Full blots can be found in supplementary Fig. 2.

### Statistical analyses

All statistical analyses were conducted using GraphPad Prism 7 software. Data were tested for normality with the Shapiro–Wilk test (*n* ≥ 3) or the D’Agostino test (*n* ≥ 8), and the appropriate test for statistical significance was applied depending on data parameters (*t* test, Mann–Whitney, one-way ANOVA with Dunnet’s post hoc or Kruskal–Wallis with Dunn’s multiple-comparison test). The tests used for each graph are outlined within the figure legends individually. Significance was at *P* value <0.05. All data points are represented as mean with standard error mean (SEM) in graphs and values stated in the text as mean with standard deviation (STDEV). In general, *n* = 3 with three to four technical replicates, details described within the figure legends for each individual data set. *F* values, *t* values and degrees of freedom (DOF) are stated within the figure legends for each set of statistical tests. Two-tailed tests for significance were used when appropriate.

## Results

### Extraction, propagation and characterisation of patient-derived CAF samples

**Fig. 2:**
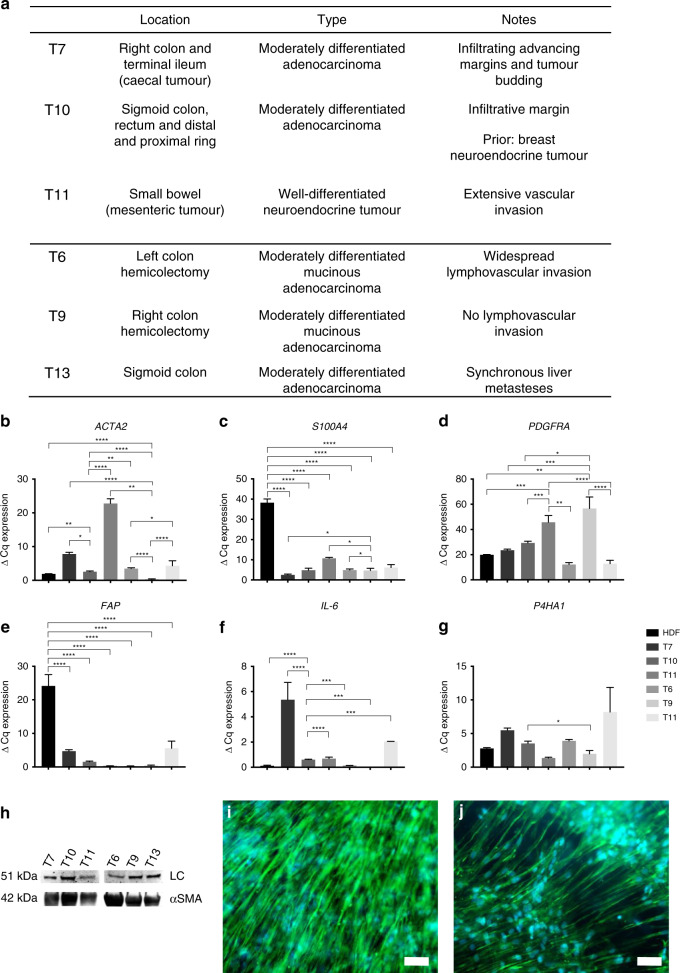
Patient-specific CAF tissue sample characterisation. **a** Origin of samples, including the location of the original cancer mass, tumour type and any additional notes. **b** 2D cell samples were analysed for fibroblast markers *ACTA2* (alpha smooth muscle actin), **c** *S100A4* (fibroblast-specific protein-1), **d** *PDGFRA* (platelet-derived growth factor receptor a), **e** *FAP* (fibroblast-activating protein), **f** *IL-6* (interleukin-6) and **g** *P4HA1* (prolyl-4-hydroxylase). The value shown is normalised to *HPRT1*mRNA levels (mean ± SEM) with *n* = 3 and three technical repeats. One-way ANOVA with Dunnet’s post hoc for *ACTA2*, *S100A4*, *FAP*, *PDGFRA* and *IL-6* and Kruskal–Wallis with Dunn’s multiple-comparison test *P* values for *P4HA1*, with values 0.05 = *, 0.005 = **, 0.0005 = *** and 0.00005 = **** with DOF for the first five genes all=20 and *F* value for *ACTA2* = 92.1, *S100A4* = 136.7, *FAP* = 31.83, *PDGFRA* = 16.2 and *IL-6* = 13.76. **h** Western blot of α-SMA protein expression within 2D monolayers of cells with LC = loading control beta-tubulin. **i**, **j** Visualisation of vimentin expression of HDF and CAFs, respectively, when cultured in 3D with scale bar = 100 µm for both images and green = vimentin and blue = DAPI.

Six patient-derived CAF samples (*n* = 6) were established from tumour samples, expanded on 2D tissue culture plastic (passage ≤ 3) and included in the tumouroid model. The samples were of variable location and origin (Fig. 2a), but all samples were from the lower bowel, colon or rectum with five being of adenocarcinoma and one being of neuroendocrine type. Samples were obtained from varying levels of tumour margin infiltration and vascular invasion. All samples were successfully cultured in 2D monolayers and tested for a number of fibroblast markers at the gene level (Fig. 2b–g). The data showed that all six samples were positive for *ACTA2*, *S100A4*, *PDGFRA, FAP, IL-6* and *P4HA1*. This confirms that the cells are activated fibroblasts, especially based on the high expression of *S100A4, PDGFRA* and *IL-6* in all samples.^26–28^ Gene expression levels were compared between the samples and HDFs and also between the different tumour fibroblast populations, which showed varying levels of expression. Secondly, the western blots showed that the α-SMA protein was expressed in all samples (Fig. 2h), this is a measure previously used to distinguish samples as CAFs.^29^ Thirdly, vimentin staining was done in CAF tumouroids grown to confluency, and the morphology was compared with normal HDFs within tumouroids (Fig. 2i, j). It was observed that the CAF samples overall appeared to have a much less-organised internal structure.

### A healthy stroma does not upregulate cancer invasion significantly

There is an evident crosstalk between the cancer cells and surrounding stroma. Within the model, two different CRC cell lines were used: the less-invasive HT29 cells and the highly invasive HCT116 cells.

**Fig. 3:**
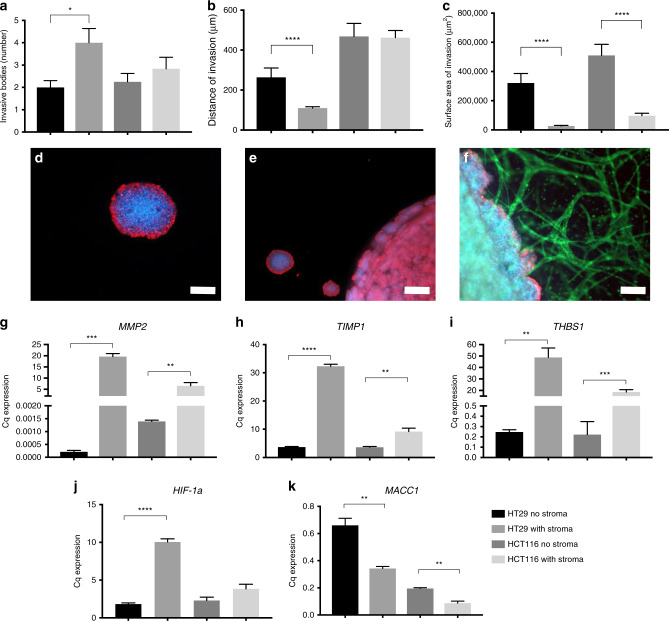
Average invasion into an acellular or healthy cellular stroma. **a** Number of invasive bodies, **b** distance of invasion and **c** average surface area of invasion at day 21 of HT29 or HCT116 tumouroids (mean ± SEM). All *n* = 3 with four technical repeats and showing Mann–Whitney *P* values, with values 0.05 = *, 0.005 = **, 0.0005 = *** and 0.00005 = ****. **d** Representative image of an invasive body, **e** invasion into acellular stroma and **f** cellular stroma within HT29 tumouroids with scale bar = 50 µm, 500 µm and 100 µm, respectively, and with red = CK20, green = CD31 and blue = DAPI. Comparative gene expression between acellular and cellular stroma in tumouroids at day 21 of growth for **g** *MMP2* (matrix metallopeptidase 2), **h** *TIMP1* (metallopeptidase inhibitor 1), **i** *THBS1* (thrombospondin 1), **j** *HIF-1α* (hypoxia-inducible factor 1-alpha) and **k** *MACC1* (metastasis associated in colon cancer-1). The value shown is normalised to *HPRT1* mRNA levels (mean ± SEM) with *n* = 3 and three technical repeats showing unpaired *t* test *P* values, with values 0.05 = *, 0.005 = **, 0.0005 = *** and 0.00005 = ****.

The effect of adding cells to the stromal compartment was measured by comparing tumouroids with an acellular stroma and tumouroids containing normal fibroblasts and primitive vascular networks (Table 2). Firstly, the number of invasive bodies increased in the presence of a cellular stroma (Fig. 3a), significantly in the HT29 tumouroids (*P* = 0.0123). However, the average distance of invasion decreased significantly (Fig. 3b) in the HT29 tumouroids (*P* = 0.0006). The surface area of invasion also decreased significantly (Fig. 3c) in the presence of a cellular stroma (*P* < 0.0001) for both the HT29 and HCT116 tumouroids. In the tumouroids, invasive bodies can be observed in the stroma (Fig. 3d, e), and extensive primitive endothelial networks are formed (Fig. 3f), whilst the fibroblast population reaches visible confluency by 21 days of growth in 3D.

**Table 2:** Average number of invasive bodies, distance of invasion and surface area of invasion within HT29 and HCT116 tumouroids containing either an acellular stroma (no stroma) or a cellular stroma (with stroma).

	Number of invasive bodies	Distance of invasion (µm)	Surface area on invasion (µm^2^)
HT29 no stroma	2.000 ± 1.044	264.3 ± 138.9	321,801 ± 182,232
HT29 with stroma	4.000 ± 2.216	110.2 ± 47.70	26,963 ± 26,871
HCT116 no stroma	2.250 ± 1.288	468.6 ± 260.8	510,240 ± 313,463
HCT116 with stroma	2.833 ± 1.801	463.0 ± 207.0	97,946 ± 95,469

When analysing the gene expression associated with the different invasion patterns, a number of genes were significantly altered when moving from an acellular to a cellular stroma, including *MMP2* for HT29 and HCT116 tumouroids (*P* = 0.001 and *P* = 0.0098, respectively), *TIMP1* (*P* ≤ 0.0001 and 0.0099, respectively) and *THBS1* (*P* = 0.0039 and *P* = 0.0007, respectively) (Fig. 3g–i).

In addition, *HIF-1a* was upregulated (Fig. 3j) in the presence of a cellular stroma compared with an acellular stroma in HT29 tumouroids (*P* ≤ 0.0001), indicating that there was more hypoxia occurring. Interestingly, *MACC1* was downregulated in the presence of a cellular stroma within the HT29 and HCT116 tumouroids (*P* = 0.0041 and 0.0024, respectively) (Fig. 3k).

### A cancerous stroma significantly upregulates cancer invasion

**Fig. 4:**
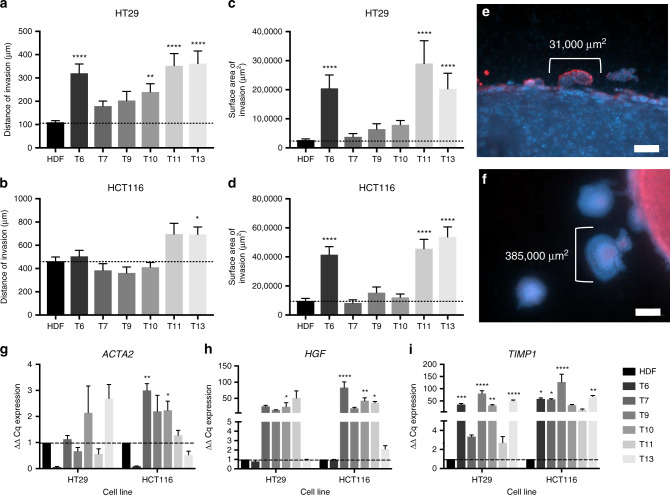
Invasion into the cancerous CAF stroma and subsequent gene upregulation. **a** HT29 distance of invasion and **b** HCT116 distance of invasion into the stroma within tumouroid models at D21 and surface are of invasion within **c** HT29 and **d** HCT116 tumouroids at D21. Tumouroids contained either HDF cells or one of six patient-specific CAF tissue samples within the stromal compartment of the constructs. All mean ± SEM with *n* = 3 with four technical repeats and showing Kruskal–Wallis with Dunn’s multiple-comparison test *P* values, with values 0.05 = *, 0.005 = **, 0.0005=*** and 0.00005 = ****. **e** Representation of average invasive bodies at day 21 in a “normal“ HDF-containing tumouroid in comparison with a (**f**) CAF-containing tumouroid. Scale bar = 100 µm for top and 500 µm for bottom image, with red = CK20 and blue = DAPI. **g** *ACTA2* (α-smooth muscle actin), **h** *HGF* (hepatocyte growth factor) and **i** *TIMP1* (metallopeptidase inhibitor 1) gene expression in tumouroids at day 21 of growth, comparing HDF- and CAF-containing stroma. The value shown is normalised to *HPRT1* mRNA levels (mean ± SEM) with *n* = 3 and three technical repeats. Ordinary one-way ANOVA Dunnett’s multiple-comparison test with *P* values 0.05 = *, 0.005 = **, 0.0005 = *** and 0.00005 = **** with DOF = 20 for all and *F* value for *ACTA2* for HT29 = 4.213 and HCT116 = 12.31, *F* value for *HGF* = 3.836 for the HT29 group and 16.3 for the HCT116 group and finally for *TIMP1* *F* value for HT29 = 37.21 and HCT116 = 11.25.

CAFs were incorporated into the 3D tumouroid model in order to investigate the effect of a cancerous stroma on cancer growth. The CAF-derived stroma caused an increase in the distance and surface area of invasion compared with the HDF-derived stroma (Fig. 4a, b, c, d and Table 3). For the less-invasive HT29^30^ tumouroids, samples T6, T10, T11 and T13 caused a significant upregulation in distance of invasion (*P* ≤ 0.0001, 0.0014, <0.0001 and <0.0001, respectively). In the highly invasive HCT116 tumouroids, CAFs statistically increased the average distance of invasion (µm) in the presence of sample T13 (*P* = 0.0489). The average surface area invaded for HT29 tumouroids was significantly greater in the presence of samples T6, T11 and T13 (*P* ≤ 0.0001 for all three). For HCT116 tumouroids, the average surface area invaded by the cancer was significantly upregulated in the presence of samples T6, T11 and T13 also (*P* ≤ 0.0001 for all three).

**Table 3:** Distance and surface area of invasion in the presence of CAFs.

	Distance of invasion (µm)		Surface area of invasion (µm^2^)	
	HT29	HCT116	HT29	HCT116
HDF	110.2 ± 47.70	463.0 ± 207.0	26,963 ± 26,871	97,946 ± 95,469
T6	320.4 ± 162.3	505.2 ± 183.7	205,140 ± 164,304	415,796 ± 181,028
T7	179.2 ± 110.7	384.5 ± 247.3	38,271 ± 51,364	83,273 ± 92,982
T9	203.7 ± 153.6	362.0 ± 222.4	64,719 ± 52,120	154,481 ± 143,668
T10	239.9 ± 132.3	411.1 ± 193.5	79,431 ± 55,283	121,401 ± 106,572
T11	352.4 ± 209.9	696.2 ± 334.9	290,460 ± 311,530	456,344 ± 232,945
T13	361.9 ± 224.0	693.6 ± 253.2	203,120 ± 193,493	537,545 ± 257,466

This is shown in the images taken by day 21 of tumouroids (Fig. 4e, f), which demonstrate the increase in size of the invasive bodies in the presence of CAFs. When comparing the effect of different cancerous stromal populations in the form of CAF samples, T11 (neuroendocrine origin) appeared to consistently cause a significant upregulation within HT29 and HCT116 tumouroids, whilst T7 on average showed the least effect (adenocarcinoma origin). A panel of 30 genes involved in invasiveness and angiogenesis were investigated to compare the healthy and cancerous stroma. Genes that were significantly upregulated in CAF tumouroids were *HGF*, *ACTA2* and *TIMP1* (Fig. 4g–i respectively, non-significant genes in Supplementary Fig. 3). In the HT29 tumouroids, *HGF* was upregulated significantly in the presence of T9 (*P* = 0.0105), and within the HCT116 tumouroids, *HGF* was significantly upregulated in the presence of samples T7 (*P* = 0.0001), T10 (*P* = 0.0071) and T11 (*P* = 0.0255). There was a tendency for increased *ACTA2* in CAF-containing HT29 tumouroids, but it was not statistically significant, whilst for the HCT116 tumouroids, *ACTA2* was upregulated significantly in the presence of samples T7 (*P* = 0.0015) and T10 (*P* = 0.0457). Finally, *TIMP1* was highly overexpressed in the presence of CAF samples. In the HT29 tumouroids, the presence of samples T6 (*P* = 0.0010), T9 (*P* = 0.0001), T10 (*P* = 0.0026) and T13 (*P* = 0.0001) significantly increased *TIMP1* expression, and in the HCT116 tumouroids, *TIMP1* expression was significantly increased in the presence of samples T6 (*P* = 0.0264), T9 (*P* = 0.0329), T10 (*P* = 0.0001) and T13 (*P* = 0.0087).

### The presence of CAFs within the tumouroid stroma inhibits vascular network formation

**Fig. 5:**
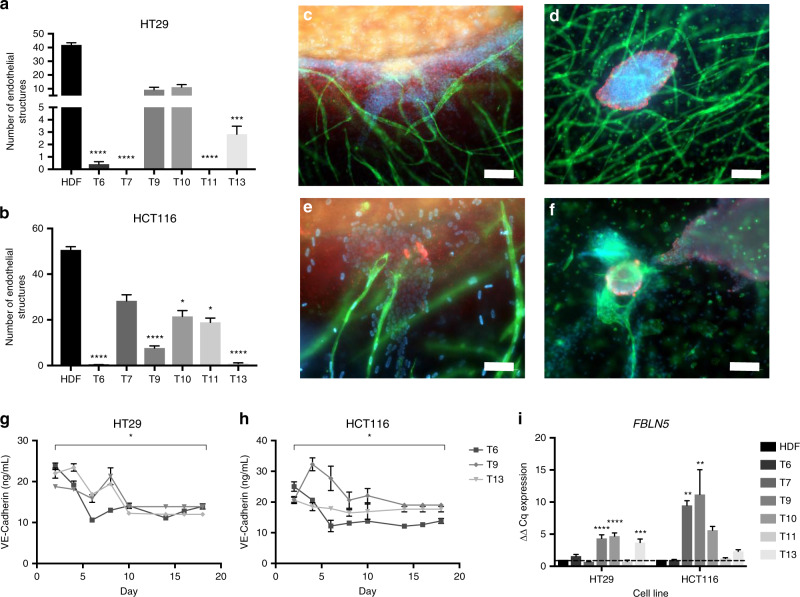
Endothelial structures formed within the cancerous CAF stroma. Number of endothelial structures formed within **a** HT29 and **b** HCT116 tumouroid stromal compartments at day 21 (mean ± SEM) containing either HDF or one of six patient-specific CAF tissue samples. All *n* = 3 with four technical repeats and showing Kruskal–Wallis with Dunn’s multiple-comparison test *P* values, with values 0.05 = *, 0.005 = **, 0.0005 = *** and 0.00005 = ****. **c** Example images of normal endothelial structure formation within a HDF-containing stroma HCT116 tumouroid at day 21 at the cancer-stromal edge and with a **d** budded invasive body within a HT29 tumouroid. Scale bar = 100 µm for the left and 50 µm for the right image, and red = CK20, green = CD31 and blue = DAPI. **e** Images showing the decreased formation of complex endothelial structures within a CAF-containing stroma at day 21 near the cancer-stroma edge, **f** as well as around a budding invasive body, the only occurrence of endothelial structures within these conditions. Scale bar = 50 µm and red = CK20, green = CD31 and blue = DAPI. Active VE-Cadherin protein released into the media of one of six CAF-containing **g** HT29 or **h** HCT116 tumouroids over 21 days (mean ± SEM). Paired *t*-test comparison test between day 2 and day 21 with *P* values 0.05 = *, 0.005 = **, 0.0005 = *** and 0.00005 = **** with DOF = 2 for both and *t* value for HT29 = 8.115 and HCT116 = 4.766. **i** *FBLN5* (fibulin-5) expression at day 21 within the HDF or one of six CAF-containing HT29 or HCT116 tumouroids (mean ± SEM). The value shown is normalised to *HPRT1* mRNA levels (mean ± SEM) with *n* = 3 and 3 technical repeats. Ordinary one-way ANOVA Dunnett’s multiple-comparison test with *P* values 0.05 = *, 0.005 = **, 0.0005 = *** and 0.00005 = ****.

CAF-containing tumouroids demonstrated an inhibition of vasculogenesis, the de novo formation of endothelial networks.^31^ This was seen as a decrease in the number of elongated endothelial structures formed within the CAF stroma by day 21 of tumouroid culture (Table 4). In the HT29 tumouroids (Fig. 5a), the number of endothelial structures was reduced significantly in the presence of samples T6, T7, T11 (all *P* ≤ 0.0001) and T13 (*P* = 0.0008) compared with endothelial structures in HDF-containing tumouroids. In the HCT116 tumouroids (Fig. 5b), the presence of CAFs significantly decreased the average number of endothelial structures for samples T6, T9 (both *P* ≤ 0.0001), T10 (*P* = 0.0479), T11 (*P* = 0.0215) and T13 (*P* ≤ 0.0001) compared with HDF- containing tumouroids.

**Table 4:** Number of endothelial structures in tumouroids.

	HT29	HCT116
HDF	41.92 ± 5.468	50.67 ± 4.697
T6	0.4167 ± 0.6686	0.2500 ± 0.4523
T7	0.00 ± 0.00	28.33 ± 9.129
T9	9.333 ± 6.213	7.667 ± 3.143
T10	11.17 ± 6.422	21.50 ± 8.990
T11	0.00 ± 0.00	18.92 ± 6.388
T13	2.833 ± 2.250	0.8333 ± 1.030

Whilst in the HDF-containing stroma, endothelial structures formed throughout the entire stromal compartment of the tumouroids (Fig. 5c, d); in the CAF-containing stroma, the formation of complex endothelial structures was only observed around invasive bodies from the cancer mass (Fig. 5e, f). The protein levels of VE-Cadherin, a protein involved in endothelial cell end-to-end fusion,^32^ showed a temporal decrease in VE-cadherin levels over the 21-day culture period. The amount of produced VE-Cadherin (ng/mL) significantly decreased in the HT29 tumouroids with sample T13 (*P* = 0.0148) from 22.07 ± 2.144 at day 2 to 12 ± 1 by day 21 (Fig. 5g). Within the HCT116 tumouroids, the VE-Cadherin production significantly decreased in the presence of T6 (*P* = 0.0413) going from 25.04 ± 2.649 at day 2 to 13.86 ± 1.415 by day 21 (Fig. 5h). The gene expression levels of *FBLN5*, a gene that inhibits endothelial cell proliferation,^33^ angiogenesis^34^ and especially sprouting,^35^ were measured in CAF- and HDF tumouroids at day 21 (Fig. 5i). In the HT29 tumouroids, samples T9, T10 (both *P* = 0.0001) and T13 (*P* = 0.0007) caused a significant upregulation in the relative gene expression, while in the HCT116 tumouroids, samples T7 (*P* = 0.0063) and T9 (*P* = 0.0014) significantly upregulated *FBLN5* compared with the HDF tumouroids.

### The disruption of preformed vascular networks by CAFs

**Fig. 6:**
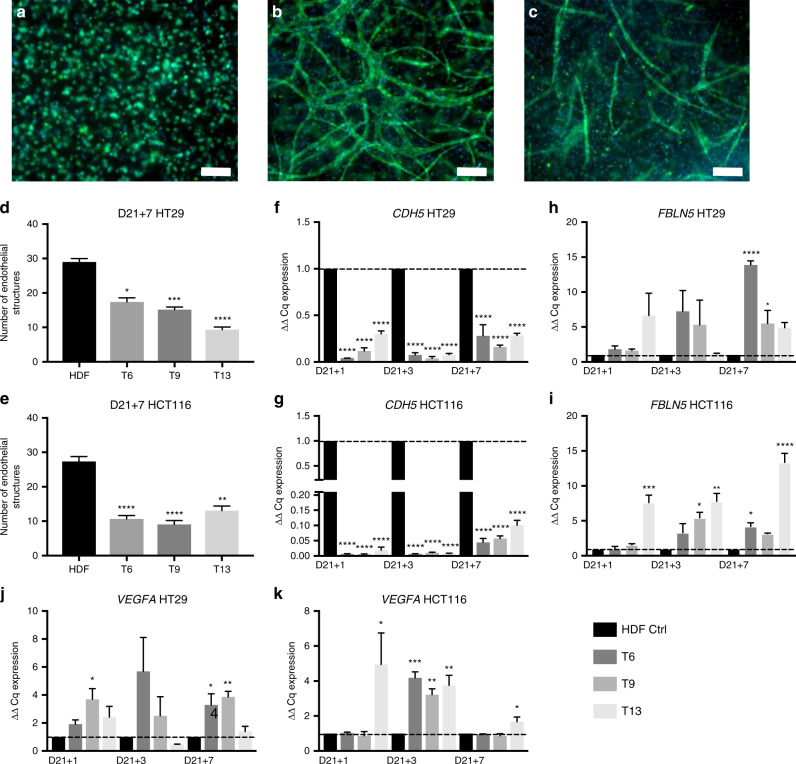
Disruption of a mature endothelial network caused by the addition of CAFs. **a** Example of single-cell endothelial cells at day 1 of tumouroid growth, **b** example of matured networks at day 21 in an HDF-containing stroma and finally **c** example of disrupted networks at day 21 + 7 (21 days normal HDF stroma growth plus 7 days post CAF addition). Scale bar = 100 µm and green = CD31 and blue = DAPI. Number of endothelial structures formed within **d** HT29 and **e** HCT116 tumouroid stromal compartments at day 21 + 7 (mean ± SEM) containing either HDF or one of six patient-specific CAF tissue samples. All *n* = 3 with four technical repeats and showing Kruskal–Wallis with Dunn’s multiple-comparison test *P* values, with values 0.05 = *, 0.005 = **, 0.0005 = *** and 0.00005 = ****. *CDH5* (VE-Cadherin) gene expression in **f** HT29 and **g** HCT116 tumouroids, *FBLN5* (fibulin-5) expression in **h** HT29 and **i** HCT116 tumouroids and *VEGFA* (vascular endothelial growth factor) expression in **j** HT29 and **k** HCT116 tumouroids after CAF addition at days 1, 3 and 7 (mean ± SEM). The value shown is normalised to *HPRT1* mRNA levels (mean ± SEM) with *n* = 3 and three technical repeats. Ordinary one-way ANOVA Dunnett’s multiple-comparison test with *P* values 0.05 = *, 0.005 = **, 0.0005 = *** and 0.00005 = ****.

In order to investigate the effect of CAFs on a developed, mature endothelial network (angiogenesis), CAF samples were added on top of tumouroids at day 21 and propagated for 7 days. Endothelial cells start of as single cells within the stroma on day 1 (Fig. 6a) and form complex, branched networks by day 21 (Fig. 6b) in the presence of HDFs within a tumouroid. After CAF addition, a disruption of the endothelial networks was observed (Fig. 6c). The disruption of vascular networks was assessed by quantifying the number and complexity of endothelial structures. The number of endothelial structures on day 21 + 7 decreased in the presence of all three CAF samples for both the HT29 and HCT116 tumouroids (Fig. 6d, e). For the HT29 tumouroids, samples T6, T9 and T13 significantly decreased the number of endothelial structures after 7 days (*P* = 0.0155, 0.0003 and <0.0001). Within the HCT116 tumouroids, samples T6, T9 and T13 also caused a significant decrease in the average number of endothelial structures after 7 days (*P* ≤ 0.0001, <0.0001 and 0.0029). The vascular disruption was further confirmed by a significant decrease in *CDH5* gene levels, coding for VE-Cadherin. Relative CDH5 levels decreased significantly (*P* < 0.0001 for all) in both HT29 and HCT116 tumouroids at day 21 + 1, day 21 + 3 and day 21 + 7 (Fig. 6f, g). Furthermore, *FBLN5* gene expression increased significantly after CAF addition (Fig. 6h, i). HT29 tumouroids contain samples T6 and T9 on day 21 + 7 (*P* = 0.0001 and 0.0395). Within the HCT116 tumouroids, *FLBN5* was upregulated significantly for T13 at day 21 + 1 (*P* = 0.0001), T9 and T13 for day 21 + 3 (*P* = 0.0261 and 0.0024) and T6 and T13 for day 21 + 7 (*P* = 0.0300 and 0.0001). Finally, *VEGFA* gene levels were analysed after CAF addition (Fig. 6J, k), and a general increase was measured. Within the HT29 tumouroids, sample T9 caused a significant upregulation at day 21 + 1 (*P* = 0.02304) and samples T6 and T9 at day 21 + 7 (*P* = 0.0233 and 0.0072). For the HCT116 tumouroids, sample T13 caused a significant upregulation at day 21 + 1 (*P* = 0.0372), samples T6, T9 and T13 at day 21 + 3 (*P* = 0.0008, 0.0071 and 0.0019) and sample T13 for day 21 + 7 (*P* = 0.0282).

## Discussion

Our findings can be summarised as follows. First, a normal healthy stroma does not upregulate cancer growth significantly in a 3D model with a defined cancer mass and defined stromal compartments. Secondly, the presence of a cancerous CAF stroma increased the distance and surface area of invasion of CRC into the stromal compartment whilst, at the same time, inhibiting vasculogenesis. These processes were associated with the upregulation of hepatocyte growth factor (*HGF*), metallopeptidase inhibitor 1 (*TIMP1*) and fibulin-5 (*FBLN5*). Next, the remodelling appeared to occur through the process of disruption of complex endothelial networks, and was associated with upregulation of vascular endothelial growth factor (*VEGFA*) and a downregulation in vascular endothelial cadherin (VE-Cadherin). These results support, within a biomimetic, 3D, in vitro framework, the direct role of CAFs in promoting cancer invasion and driving both vasculogenesis and angiogenesis.

We did observe an increase in the number of invasive bodies from the cancer mass into the healthy stroma. This models how highly invasive cancers “disperse” into a high number of small clusters to invade the surrounding stromal tissue. This is often due to the loss of structural proteins, such as cadherins and cytokeratins.^36^

The second major finding of this novel work is the differential invasion rate and pattern of less-invasive HT29 and highly invasive HCT116 cancer cells in 3D tumouroids in the presence of CAFs, causing an increase in the distance (up to threefold) and surface area of invasion (up to tenfold) over a 21-day period. CAF pro-invasive properties and their ability to enable cancer cells to metastasise has been demonstrated previously.^37,38^ Logsdon et al.^39^ described the importance of CAFs in a 3D pancreatic cancer model using Matrigel^®^-coated invasion chambers and soft-agar colony formation, and although this model showed an increase in proliferation and metastasis during in vivo validation, the 3D model was not compartmentalised and did not allow for a measurement of invasion in vitro. The majority of 3D cancer models lack appropriate tensile force and stiffness associated with tumour tissue, as they commonly use soft hydrogels, which have too high a water content.^40^ Our biomimetic 3D in vitro cancer model (tumouroid) has a collagen density of up to 40× higher compared with standard hydrogels, and therefore mimics the in vivo stiff tumour environment more closely,^20^ an important aspect especially for CAFs.^41^

The third of our observations can be interpreted in the following manner: CAFs play a key role in vascular network formation and remodelling. Whilst it is understood that CAFs play a major role in angiogenesis and recruiting vasculature towards the cancer,^27^ in this study, we also demonstrated that CAFs play a major role in vasculogenesis and the disruption of vascular network formation. This aspect has not been studied with the same rigour. Some studies have introduced CAFs at the same time point as HUVECs and observed end-to-end fusion of the HUVEC cells into endothelial structures.^42^ Whilst this could be an observation of vasculogenesis, our model, with tissue-specific parameters, including biomimetic matrix density, shows no de novo formation of vascular networks in 3D in the presence of CAFs. At 25 000 CAFs per 24-well tumouroids, we observed 100% confluency of these cells in 3D by day 7, whilst HUVECs in our “normal” HDF-containing cultures would not start forming complex endothelial structures until at least day 14. Our data indicate that CAFs start expressing factors that block complex vascular network formation, whilst retaining “simple” vascular/endothelial structures. One of the factors, that was significantly increased, was *VEGFA*. Although *VEGFA* is the major player in angiogenesis and involved in recruiting mature blood vessels towards the cancer, its role in vasculogenesis is not as well understood. The major crosstalk between cancer cells and endothelial cells in our set-up was growth factor driven, which was ascertained through an additional 3D set-up. This set-up demonstrated the chemoattractant-driven movement and recruitment of endothelial structures to the cancer mass through an acellular ring placed between the cancer mass and stromal compartment.^19^ Interestingly, a study looking at cardiac mouse development found a correlation between the disruption of vasculogensis and elevated *VEFGA* levels.^43^ This aspect could be observed within our work as the cancer cells and CAFs co-evolve during tumour progression. This was further studied by Brown et al. when looking at how the prevention of vasculogenesis but not angiogenesis prevented the recurrence of glioblastoma in mice.^44^ Cancer cells are known for their high turnover and overproduction of angiogenic growth factors.^45^ This is in an attempt to recruit host vasculature from surrounding tissues. The unregulated and upregulated production and release of angiogenic growth factors by solid tumours results in the formation of aberrant and leaky vasculature surrounding tumours.^46^ We have measured increased levels of VEGFA in our tumouroid cultures, which are resulting in disrupted vasculogenesis and angiogenic remodelling to form non-complex networks. Vasculogenesis in cancer and especially in relation to the presence of CAFs is not a major focus of research as angiogenesis and remodelling of cancer is the biomimetic environment in which cancer arises. However, by gaining insights into how cancer angiogenic signalling can influence vasculogenesis may help further our understanding of tissue necrosis and vascular remodelling in cancer.

We further showed that CAFs have the ability to disrupt preformed in vitro vascular networks (“CAF treatment”). By day 7, post CAF addition, the endothelial networks that had previously formed were disrupted, and an overall decrease in the number of endothelial structures was observed. Furthermore, we found a decrease of *CDH5* levels within the tumouroids. The role of *CDH5* and the corresponding protein VE-Cadherin is becoming more pertinent in the study of angiogenesis as it has been specifically implicated in the local production of junctions within complex endothelial networks.^32^ The literature on CAF interaction with VE-Cadherin is limited, although the role of CAFs as major sources of VEGFA production is established and understood to be mediated through HIF-1α/GPER signalling.^47^ This particular gene (*VEGFA*) was increased after we added CAFs to our cultures, and in fact, this will have played an important role in the disruption (or angiogenesis) observed; however, it would be crucial to study further how stromal cells cause this angiogenesis as the normalisation of these remodelled vascular networks has been a target for many antiangiogenic drugs.^48^

Our results need to be understood in the context of the following methodological limitations. We report the interactions at the interface of a cancer mass and patient-derived cancer-associated fibroblasts in a 3D-vascularised colorectal cancer model. A total of six patient-derived CAF samples were successfully isolated and cultured from colorectal cancer tissue samples. Primary CAF characterisation is a topic of debate in literature. Some groups have done extensive characterisation on the gene and protein level of “CAF specific” markers.^27,49,50^ In this study, we successfully demonstrated that our CAF samples expressed widely recognised markers for CAF identification. In the past, CAF populations were often classified purely based on their location within the tumour margin. For comparison purposes, we used human dermal fibroblasts (HDFs) as our control or “healthy” stromal cells, which are not an immortalised cell line and could also start to differentiate into CAFs while co-cultured with cancer cells within the tumouroids. It could be argued that most primary fibroblast samples will adapt a cancerous phenotype due to being cultured on plastic or in co-culture with cancer cells.^51^ For future work, it would be ideal to use paired CAF and NF samples from the same patient as part of a larger comparison study. Patient-derived NFs would serve as a better control and further our investigations into what signalling is caused by CAFs. In addition, following on from this work, potential of identifying what drives CAFs and their cancer-promoting properties could be pinpointed. One pathway would be to generate knockout CAFs with a deletion of *HGF*, *TIMP1* and *FBLN5* genes we found to be upregulated in the tumouroids and possibly responsible for increased invasion and vascular remodelling. For example, the inhibition of the HGF/c-Met signalling pathway is a compelling therapy to interfere with tumour growth and angiogenesis.^52^ Specific protagonists to VE-Cadherin could be used to potentially normalise the “leaky vasculature” caused by VEGFA upregulation, which are independent of one another. Along the lines of gene expression analysis, a major limitation within this study is the use of the whole, multicellular tumouroid, which does not allow for the analysis of single-cell signalling. This calls for the data presented in this work to be called “observational” highlighting correlations, not causations. Overall, we believe this piece of work has furthered our understanding of the interactions between cancer cells and the stroma, healthy or cancerous. This lays the groundwork for future works further investigating the reactive cancer stroma within a sophisticated 3D model set-up.

## Supplementary information

Supplementary Section

## Data Availability

The data that support the findings of this study are available from the corresponding author (U.C.) on reasonable request.
