# Supplementary material for: Cancer-associated fibroblasts mediate cancer progression and remodel the tumouroid stroma
Source: Br J Cancer. 2020 Jul 9;123(7):1178–90. doi: 10.1038/s41416-020-0973-9 (PMC7524802; doi:10.1038/s41416-020-0973-9)
Supplement: Supplementary file 1 — Supplementary Section [file 41416_2020_973_MOESM1_ESM.pdf]

**Supplementry Section**

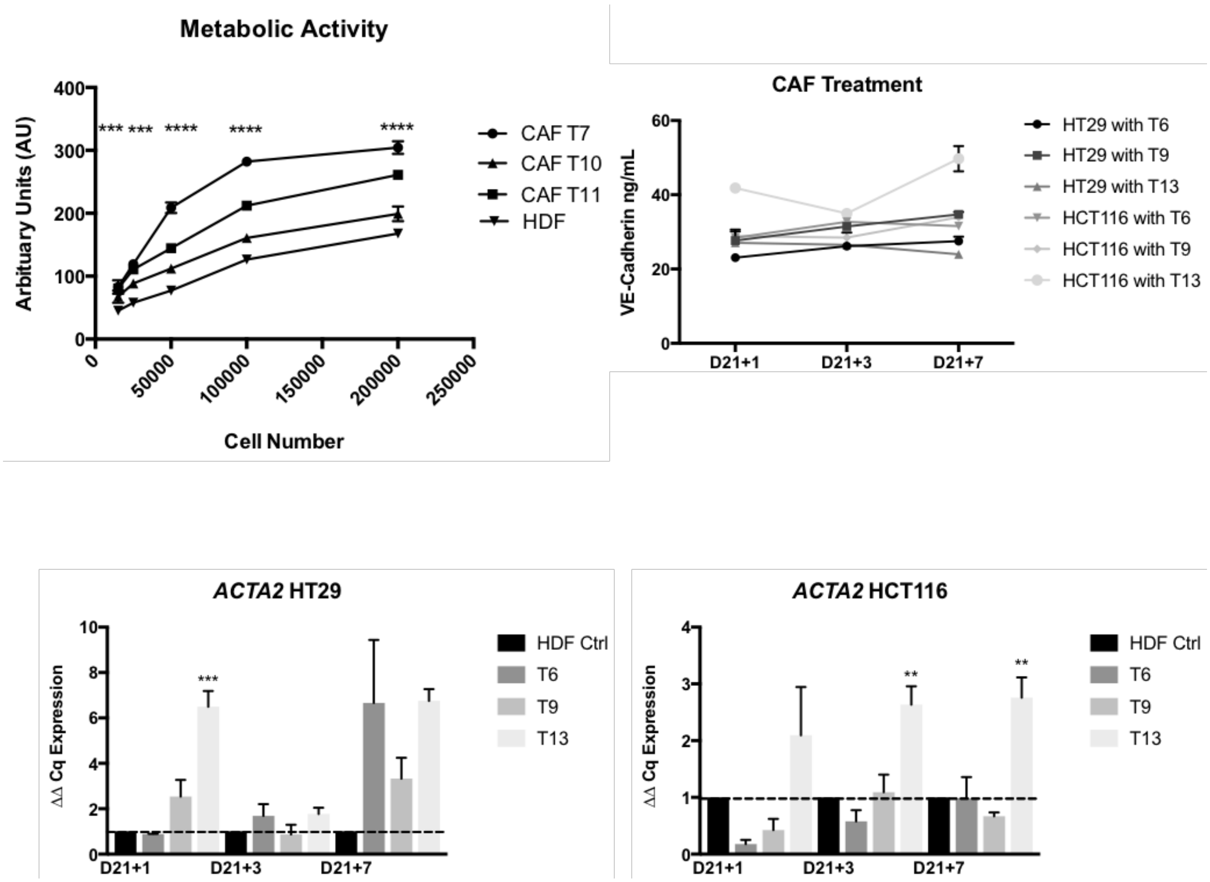


*Figure 1: CAF metabolic activity and "CAF treatment". Metabolic activity of CAF samples compared to HDFs. VE-Cadherin protein expressed into media after "CAF treatment". ACTA2 gene levels in tumouroids after "CAF treatment" for HT29 and HCT116 tumouroids.*

**Primer design parameters**

Design in CDS- preferably towards 3' end if possible and to be exon spanning. Product Size: Min-100bp Opt-120bp Max- 200bp. Primer Size: Min-20bp Opt-22bp Max-25bp. Primer Tm: Min- 58°C Opt-60°C Max-65°C (so that Ta will be ca. 60°C). Max Self Complementary: 5,00. Max 3'Self Complemtarity: 3,00. Max. Poly-X: 3. GC Clamp: 1. Use mfold (http://mfold.rna.albany.edu/?q=mfold/DNA-Folding-Form) to check for secondary structures. Folding Temperature: 60°C. Ionic Conditions of SYRB in mM: 50$Na^+$, 3$Mg^{2+}$. Structures: few loops, best if none, no loops in primer attachment region, ΔG ok until -4. Use Primer BLAST to check for specificity.

Alpha smooth muscle actin full western blot

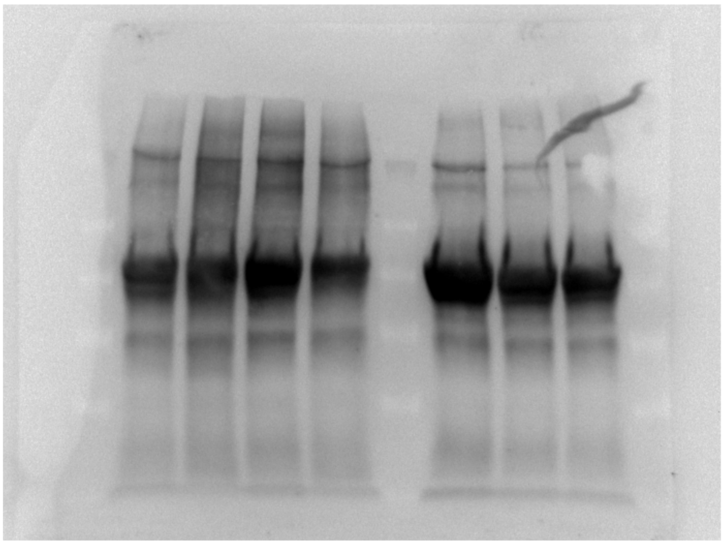

Beta tubulin loading control full western blot

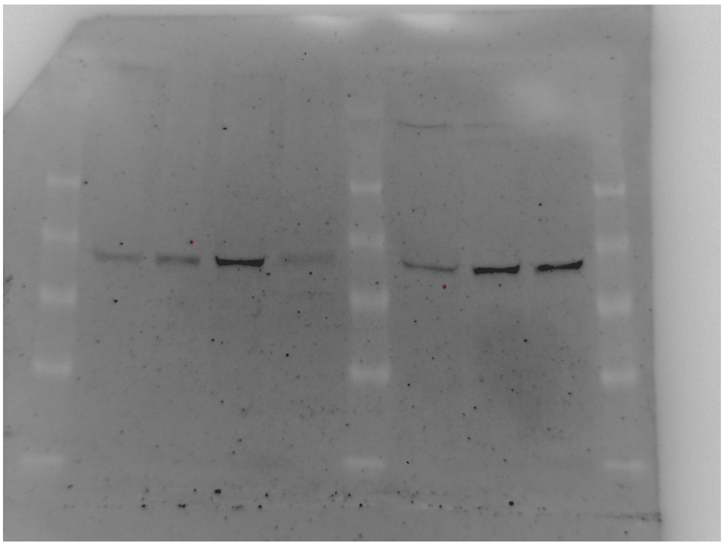

*Figure 2: Full western blots for alpha smooth muscle actin and beta tubulin for HDF and CAF samples.*

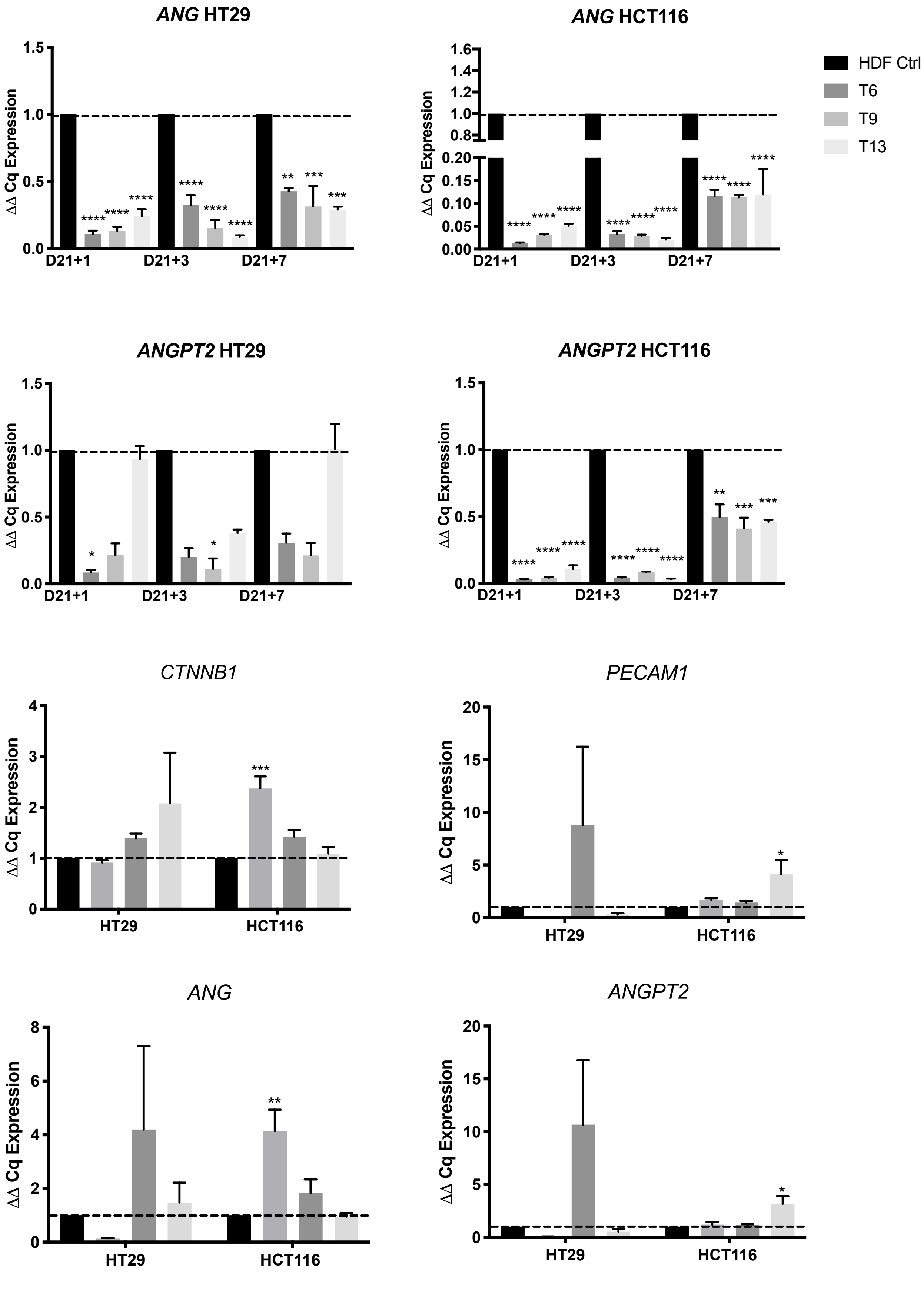

ANG HT29
ANG HCT116
ANGPT2 HT29
ANGPT2 HCT116
CTNNB1
PECAM1
ANG
ANGPT2
ΔΔ Cq Expression
D21+1
D21+3
D21+7
HT29
HCT116
HDF Ctrl
T6
T9
T13

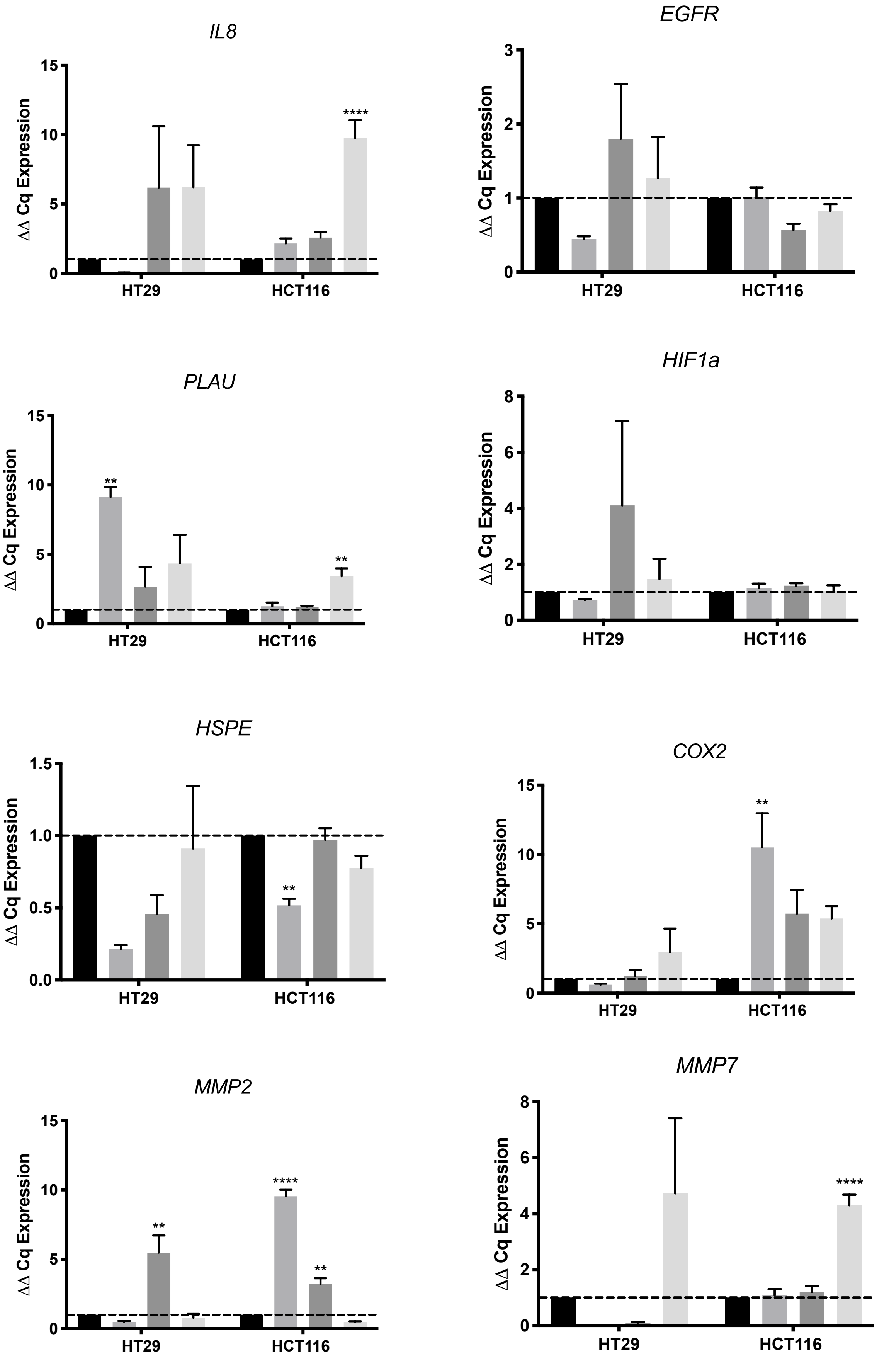
IL8
ΔΔ Cq Expression
15
10
5
0
****
HT29
HCT116
EGFR
ΔΔ Cq Expression
3
2
1
0
HT29
HCT116
PLAU
ΔΔ Cq Expression
15
10
5
0
**
**
HT29
HCT116
HIF1a
ΔΔ Cq Expression
8
6
4
2
0
HT29
HCT116
HSPE
ΔΔ Cq Expression
1.5
1.0
0.5
0.0
**
HT29
HCT116
COX2
ΔΔ Cq Expression
15
10
5
0
**
HT29
HCT116
MMP2
ΔΔ Cq Expression
15
10
5
0
**
****
**
HT29
HCT116
MMP7
ΔΔ Cq Expression
8
6
4
2
0
****
HT29
HCT116

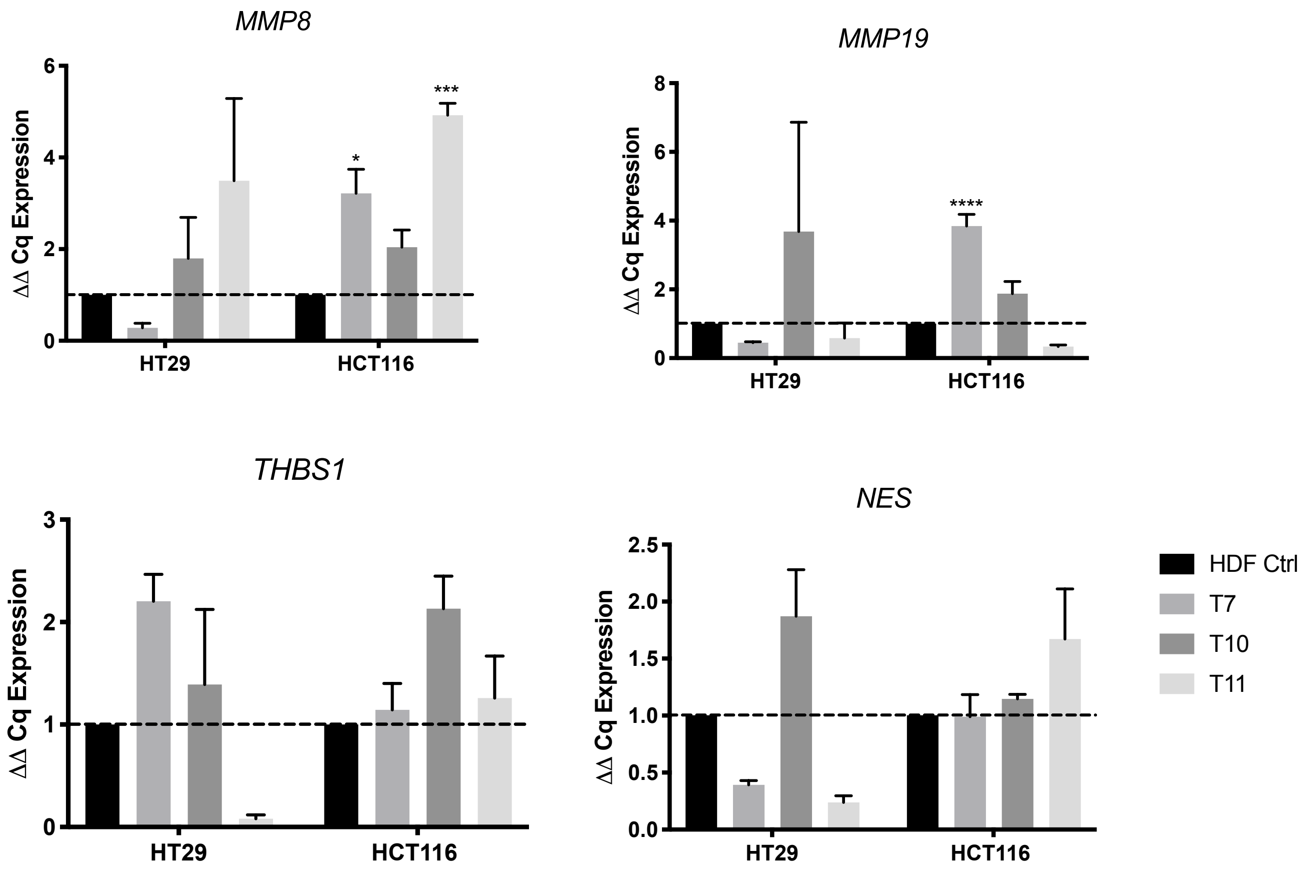


*Figure 3: Additional genes tested on CAF samples from primer Table 1*
